# Effect of comorbid mood and anxiety disorders on breast and cervical cancer screening in immune-mediated inflammatory disease

**DOI:** 10.1371/journal.pone.0249809

**Published:** 2021-08-05

**Authors:** Ruth Ann Marrie, Randy Walld, James M. Bolton, Jitender Sareen, Scott B. Patten, Alexander Singer, Lisa M. Lix, Carol A. Hitchon, James J. Marriott, Renée El-Gabalawy, Alan Katz, John D. Fisk, Charles N. Bernstein, for the CIHR Team in Defining the Burden and Managing the Effects of Psychiatric Comorbidity in Chronic Immunoinflammatory Disease

**Affiliations:** 1 Department of Internal Medicine, Max Rady College of Medicine, Rady Faculty of Health Sciences, University of Manitoba, Winnipeg, Canada; 2 Department of Community Health Sciences, Max Rady College of Medicine, Rady Faculty of Health Sciences, University of Manitoba, Winnipeg, Canada; 3 Manitoba Centre for Health Policy, Max Rady College of Medicine, Rady Faculty of Health Sciences, University of Manitoba, Winnipeg, Canada; 4 Department of Psychiatry, Max Rady College of Medicine, Rady Faculty of Health Sciences, University of Manitoba, Winnipeg, Canada; 5 Department of Community Health Sciences, Cumming School of Medicine, University of Calgary, Calgary, Canada; 6 Department of Family Medicine, Max Rady College of Medicine, Rady Faculty of Health Sciences, University of Manitoba, Winnipeg, Canada; 7 Department of Clinical Health Psychology, Max Rady College of Medicine, Rady Faculty of Health Sciences, University of Manitoba, Winnipeg, Canada; 8 Department of Anesthesia and Perioperative Medicine, Max Rady College of Medicine, Rady Faculty of Health Sciences, University of Manitoba, Winnipeg, Canada; 9 Nova Scotia Health Authority and the Departments of Psychiatry, Psychology & Neuroscience, and Medicine, Dalhousie University, Halifax, Canada; Ordu University, TURKEY

## Abstract

We aimed to examine rates of breast and cervical cancer screening in women with immune-mediated inflammatory diseases (IMID), including inflammatory bowel disease (IBD), multiple sclerosis (MS) and rheumatoid arthritis (RA) versus a matched cohort with IMID; and examine the association of psychiatric comorbidity with screening in these populations. We conducted a retrospective cohort study in Manitoba, Canada using administrative data. We identified women with IBD, MS and RA, and controls without these IMID matched on age and region. Annually, we identified individuals with any active mood/anxiety disorder. Using physician claims, we determined the proportion of each cohort who had cervical cancer screening within three-year intervals, and mammography screening within two-year intervals. We modeled the difference in the proportion of the IMID and matched cohorts who underwent mammography; and pap tests using log-binomial regression with generalized estimating equations, adjusting for sociodemographics, comorbidity and immune therapy use. We tested for additive interactions between cohort and mood/anxiety disorder status. During 2006–2016, we identified 17,230 women with IMID (4,623 with IBD, 3,399 with MS, and 9,458 with RA) and 85,349 matched controls. Having an IMID was associated with lower (-1%) use of mammography; however, this reflected a mixture of more mammography in the IBD cohort (+2.9%) and less mammography in the MS (-4.8 to -5.2%) and RA (-1.5%) cohorts. Within the IBD, MS and RA cohorts, having an active mood/anxiety disorder was associated with more mammography use than having an inactive mood/anxiety disorder. The MS and RA cohorts were less likely to undergo Pap testing than their matched cohorts. In the absence of an active mood/anxiety disorder, the IBD cohort was more likely to undergo Pap testing than its matched cohort; the opposite was true when an active mood/anxiety disorder was present. Among women with an IMID, mood/anxiety disorder influence participation in cancer screening.

## Introduction

Preventive care, such as cancer screening and immunizations, is important for prevention and early identification of potentially life-threatening disease. However, persons with chronic conditions may have lower rates of preventive care than the general population. One study of persons over age 65 years in Ontario, Canada suggested that the presence of one chronic disease reduced the likelihood of treatment for unrelated chronic diseases and that the use of disease-modifying (immune) therapies and presence of psychotic disorders, in particular, reduced the likelihood of treatment [1]. The influence of immune-mediated inflammatory disease (IMID), such as inflammatory bowel disease (IBD), multiple sclerosis (MS) and rheumatoid arthritis (RA), on use of preventive care is poorly understood but this knowledge is important to inform interventions that support preventive care in clinical practice in these populations. Prior studies have had conflicting findings [2, 3]. Moreover, these studies often included small samples, lacked control groups to account for use of preventive care in the general population, or were not recent enough to reflect current practice [3, 4].

Several factors may influence uptake of preventive care in persons with IMID. As health care utilization is elevated in persons with IMID as compared to those without IMID [5, 6], this could increase the access to, or consideration of preventive care. However, IMID are associated with physical and cognitive impairments that may create barriers to preventive care [7]. Mobility impairments are a particular concern in MS and RA and women with MS who have mobility impairments are less likely to undergo cancer screening than women with MS without mobility impairments [8, 9]. Also, comorbidity is common in IMID, particularly psychiatric comorbidity which occurs 1.5 to 2-fold more often in persons with IMID than without IMID. Among women, in Ontario, Canada, the likelihood of cancer screening was found to decrease with an increasing number of comorbidities, and the likelihood of cervical cancer screening decreased with increasing comorbidities and increasing level of disability [10]. In other populations, psychiatric disorders have been associated with reduced use of preventive services [11–13]. The influence of psychiatric disorders on cancer screening in persons with IMID is unknown, but this is important given that psychiatric disorders are common and treatable.

We aimed to determine if women with IBD, MS and RA had differential use of preventive care relative to a matched general population cohort, and whether their preventive care use was influenced by psychiatric comorbidity. Specifically, we examined cervical cancer screening, and mammography [14]. We focused on these screening tests because of the predominance of women in the MS and RA populations, and the potentially strong barrier imposed by physical impairments on cervical and breast cancer screening. Further, these screening tests can be tracked using administrative health data. We reasoned that if the effects of IMID and psychiatric comorbidity on preventive care are consistent across three IMID affecting different organ systems and causing differing physical impairments, then the effects would be likely to generalize to other IMID. We hypothesized that persons with IMID would have lower rates of preventive care than persons without IMID, and that the presence of psychiatric comorbidity would further reduce preventive care. Also, we aimed to determine the within-person effects of psychiatric comorbidity on cervical cancer screening and mammography. Specifically, for each person who ever met the case definition for a psychiatric disorder based on administrative health data, annually we determined whether the condition was active or inactive [15]. We reasoned that the effects of a psychiatric disorder would occur mainly when the disorder was active (symptomatic) [16–18], potentially due to factors such as lack of interest, suicidal ideation, avoidance, cognitive effects, or other mechanisms.

## Methods

We conducted this population-based retrospective matched cohort study in Manitoba, Canada using secondary analysis of administrative health data. Manitoba has a population of approximately 1.3 million and medically necessary health care services are universal and publicly funded. Health services used are prospectively recorded in administrative databases. Each Manitoba resident has a unique personal health identification number which is attached to the records of health services. The University of Manitoba Health Research Ethics Board approved the study, and Manitoba’s Health Information Privacy Committee approved access to administrative (health claims) data.

### Data sources

We used administrative (health claims) databases in the Population Research Data Repository at the Manitoba Centre for Health Policy including the population registry, Discharge Abstract Database, medical services (physician claims) database, and Drug Programs Information Network database. The population registry provided information regarding dates of birth, death and health care coverage; sex; and region of residence (postal code). The Discharge Abstract Database provided information regarding hospitalizations including admission and separation dates and diagnostic codes. Diagnoses were recorded using the International Classification of Disease 9^th^ revision clinical modification (ICD-9-CDM) before April 2004, and by ICD 10^th^ revision, Canadian version (ICD-10-CA) thereafter, with up to 25 diagnosis codes recorded for each hospitalization after April 2004 and up to 18 diagnosis codes before this date. The medical services database provided date of service and one physician-assigned diagnosis recorded using ICD-9-CM codes. The Drug Programs Information Network records prescriptions dispensed in the community, including the drug identification number and name, and dispensation date. We accessed these databases for the period April 1, 1984 to March 31, 2016, except for Drug Programs Information Network, which was available from 1995 onward.

### Study populations

We applied validated case definitions to identify Manitobans with IBD, MS and RA [19–21], during the period 1984–2016 as delineated elsewhere [22]. For each condition, the first relevant health claim was designated as the index date. Next, we identified a cohort from the general population, which excluded anyone with ICD-9-CM/ICD-10-CA codes for IBD, MS, or RA, or use of MS-specific disease modifying therapies included in the case definition for MS. Within this cohort we randomly selected 5 controls for each case, matched with birth year ± 5 years, sex, and forward sortation area (first three digits of postal code). Each control was assigned the index date of their matched case. Then, the IMID and matched cohorts were limited to women aged ≥18 years at the index date. Finally, to reduce complexity related to secular changes in the data and screening guidelines, we limited the study population (which had been created to support several studies) [22] to prevalent cases and controls alive during the period 2006–2016.

### Psychiatric morbidity

Subsequently, we applied validated case definitions for depression, anxiety, and any mood or anxiety disorder (which included ≥1 of depression, anxiety disorders or bipolar disorders) [23], to identify these conditions in our study cohorts [23, 24]. The first hospital or physician claim for each condition was designated as the index date. We considered the date of the first claim for each condition to be the date of diagnosis. Since psychiatric disorders can relapse and remit [25–27], and health care use may fluctuate depending on psychiatric status, we reassessed psychiatric status annually, as described previously [15]. Specifically, after an individual met the case definition for any mood/anxiety disorder, the individual was considered to be an annual prevalent (‘active’) case if there were ≥2 physician claims or one hospital claim with a diagnostic code for the psychiatric disorder in that year; for hospital claims the psychiatric disorder was required to be the most responsible diagnosis [15]. Prescription claims alone were not considered as a marker of ‘active’ disease due to their potentially frequent off-label use in MS populations. Therefore, psychiatric status could vary from ‘active’ to ‘inactive’ each year.

### Preventive health care use

In Manitoba, population-based breast cancer screening is offered starting at age 50 years when women are invited by letter to book an appointment for a mammogram. A recall invitation letter is sent every two years. Mammograms may also be ordered by physicians if patients did not respond to the invitation letter for the provincial screening program. Cervical cancer screening is recommended every three years but relies on the patient to book an appointment with their primary care provider or gynecologist or a Papanicolaou (Pap) test clinic. Primary care providers may also recommend testing in the course of usual care or via standard office practices (i.e. patient recalls for health review). Using the specific tariff (i.e., service) codes from provider claims we identified Pap smears (8470, 8495, 8496, 8498, 9795) and mammograms (7098, 7099, 7104, 7110, 7111). For Pap smears, we included women aged 25–69, and considered one Pap test in a three-year period to indicate screening had occurred. For mammograms, we included women aged 50–69 years, and we considered one mammogram in a two-year period to indicate screening had occurred. These age groups were chosen to reflect those for whom screening was recommended in Manitoba throughout the entire study period.

### Covariates

Covariates included in multivariable analyses included sociodemographic characteristics, physical comorbidities and use of immune therapies. The sociodemographic characteristics included age (mammograms: 50–59 [reference], 60–69 years; Pap test: 25–34 [reference], 35–44, 45–54, 55–69 years), socioeconomic status in quintiles (lowest quintile of SES as reference group), and region (urban or rural [reference group]). We derived socioeconomic status by linking postal code to dissemination-area level census data, then calculating the Socioeconomic Factor Index version 2 (SEFI-2) which incorporates information regarding high school education rate, average household income, unemployment rate and percent of single parent households; scores less than zero indicate higher socioeconomic status [28]. We classified Winnipeg (population>600,000) and Brandon (population>47,000) as urban regions, and all other regions as rural. To measure physical comorbidity, we used the John Hopkins Adjusted Clinical Group System to identify Aggregated Diagnosis Groups (ADGs), targeting major physical ADGs that were chronic (ADGs 9, 11, 16, 22 and 32); these were summarized as 0, 1 or ≥2 ADGs.

We included year of diagnosis and disease duration as continuous variables. As a measure of disease severity, we included IMID-specific procedures. For IBD these included surgical procedures related to gastrointestinal resections or ostomy placement (S1 Table), and for RA these included joint-related surgical procedures (S2 Table); there were no relevant procedures for MS. We categorized immune therapies for IBD and RA as none (reference group), any biologic (alone or in combination), or any anti-inflammatory or traditional immunosuppressive therapy or corticosteroids. For MS, we categorized immune therapies as none, first-line, or second-line (S3 Table).

### Analysis

We characterized the study cohorts using descriptive statistics including mean (standard deviation [SD]), median (interquartile range [IQR]), and frequency (percent [%]). For each cohort, we report the crude percentage (95% confidence interval [95%CI] based on a binomial distribution) who received screening, as well as the percentages, which were age-standardized to the 2010 Canadian population.

We modeled the absolute, instead of relative, difference in proportions screened according to cohort (combined IMID vs. matched), in the year after determination of mood/anxiety disorder status (active vs. inactive) using log-binomial regression models with generalized estimating equations with an exchangeable correlation coefficient to account for within-person correlation. We accounted for differences in follow-up time by including the log of person-years as the model offset. These linear models provide population averages of within-person and between-person effects, but we parameterized them to separate these effects using a person-mean variable and a within-person centered variable [29]. We tested for the presence of additive interactions between cohort and mood/anxiety disorder status. A positive (synergistic) interaction would indicate the joint effects of cohort and mood/anxiety disorder exceeded the sum of their individual effects. A negative interaction would indicate the joint effect was less than the sum of their individual effects. We repeated these analyses separately for each IMID (IBD, MS, RA) cohort.

Statistical analyses were conducted using SAS V9.4 (SAS Institute Inc., Cary NC).

## Results

### Study populations

During the period 2006–2016, we identified 17,230 women with IMID, including 4,623 with IBD, 3,399 with MS, and 9,458 with RA, as well as 85,349 matched controls. Due to differential rates of deaths before 2006, 11,527 matched controls no longer had matched cases, and only 10,324 IMID cases had 5 controls. Nonetheless, the IMID and matched cohorts remained well-matched with respect to the matching variables of age and region (Table 1). Two-thirds of the IMID and matched cohorts lived in urban regions, and one-third of the IMID cohort had one or more physical comorbidities. At the index date, nearly 40% of the IMID cohort had a lifetime history of any mood/anxiety disorder, 10% more than the matched cohort. The prevalence of any ‘active’ mood/anxiety disorder was also greater in the IMID cohort than the matched cohort.

**Table 1:** Characteristics of prevalent disease cohorts and matched cohorts.

Characteristic	IMID matches (n = 85349)	IMID (n = 17230)	IBD matches (n = 22207)	IBD (n = 4623)	MS matches (n = 17247)	MS (n = 3399)	RA matches (n = 47102)	RA (n = 9458)
Age at diagnosis/index date^a^ (years), mean (SD)	46.5 (15.9)	46.0 (16.0)	41.7 (16.6)	41.3 (16.6)	40.1 (11.9)	39.5 (11.6)	51.2 (15.4)	50.7 (15.5)
Urban region of residence, n (%)	53263 (62.4)	10804 (62.7)	14656 (66.0)	3048 (65.9)	11479 (66.6)	2279 (67.0)	27919 (59.3)	5645 (59.7)
Socioeconomic status, mean (SD)	-0.06 (0.97)	-0.08 (0.99)	-0.24 (0.89)	-0.21 (0.86)	-0.22 (0.85)	-0.25 (0.88)	0.07 (1.0)	0.05 (1.1)
Physical Comorbidity (ADGs), n (%)								
0	70989 (83.2)	11881 (69.0)	18824 (84.8)	3058 (66.1)	15035 (87.2)	2489 (73.2)	38078 (80.8)	6390 (67.6)
1	12461 (14.6)	4377 (25.4)	2951 (13.3)	1289 (27.9)	1985 (11.5)	736 (21.7)	7747 (16.4)	2511 (26.5)
≥2	1899 (2.2)	972 (5.6)	432 (1.9)	276 (6.0)	227 (1.3)	174 (5.1)	1277 (2.7)	557 (5.9)
Psychiatric comorbidity, n (%)								
Any Psychiatric Disorder	25096 (29.4)	6883 (40.0)	6337 (28.5)	1814 (39.2)	5030 (29.2)	1553 (45.7)	14137 (30.0)	3664 (38.7)
Any Mood or Anxiety Disorder	24791 (29.1)	6822 (39.6)	6277 (28.3)	1805 (39.0)	4992 (28.9)	1541 (45.3)	13927 (29.6)	3624 (38.3)
Depression	21582 (25.3)	5902 (34.3)	5543 (25.0)	1597 (34.5)	4427 (25.7)	1365 (40.2)	11968 (25.4)	3077 (32.5)
Anxiety	30378 (35.6)	7382 (42.8)	7594 (34.2)	1970 (42.6)	6051 (35.1)	1534 (45.1)	17209 (36.5)	4026 (42.6)
Bipolar disorder	3545 (4.2)	988 (5.7)	902 (4.1)	326 (7.1)	797 (4.6)	234 (6.9)	1910 (4.1)	452 (4.8)
Active prevalence any mood or anxiety disorder	6933 (8.1)	1901 (11.0)	1857 (8.4)	564 (12.2)	1450 (8.4)	442 (13.0)	3742 (7.9)	933 (9.9)
Active prevalence depression	6449 (7.6)	1765 (10.2)	1733 (7.8)	546 (11.8)	1352 (7.8)	420 (12.4)	3468 (7.4)	838 (8.9)
Active prevalence anxiety	4332 (5.1)	1121 (6.5)	1197 (5.4)	344 (7.4)	891 (5.2)	259 (7.6)	2311 (4.9)	540 (8.9)
Active prevalence bipolar	837 (0.98)	196 (1.1)	225 (1.0)	73 (1.6)	213 (1.2)	54 (1.6)	415 (0.88)	74 (0.78)

IBD = inflammatory bowel disease, MS = multiple sclerosis, RA = rheumatoid arthritis, IMID = immune-mediated inflammatory disease, combining IBD, MS and RA cohorts; Socioeconomic status = Socioeconomic Factor Index scores; values less than zero indicate higher socioeconomic status; a- for IMID, the date of diagnosis = their index date; controls were assigned the same index date as their matched cases.

### Mammograms in IMID cohort

**Fig 1:**
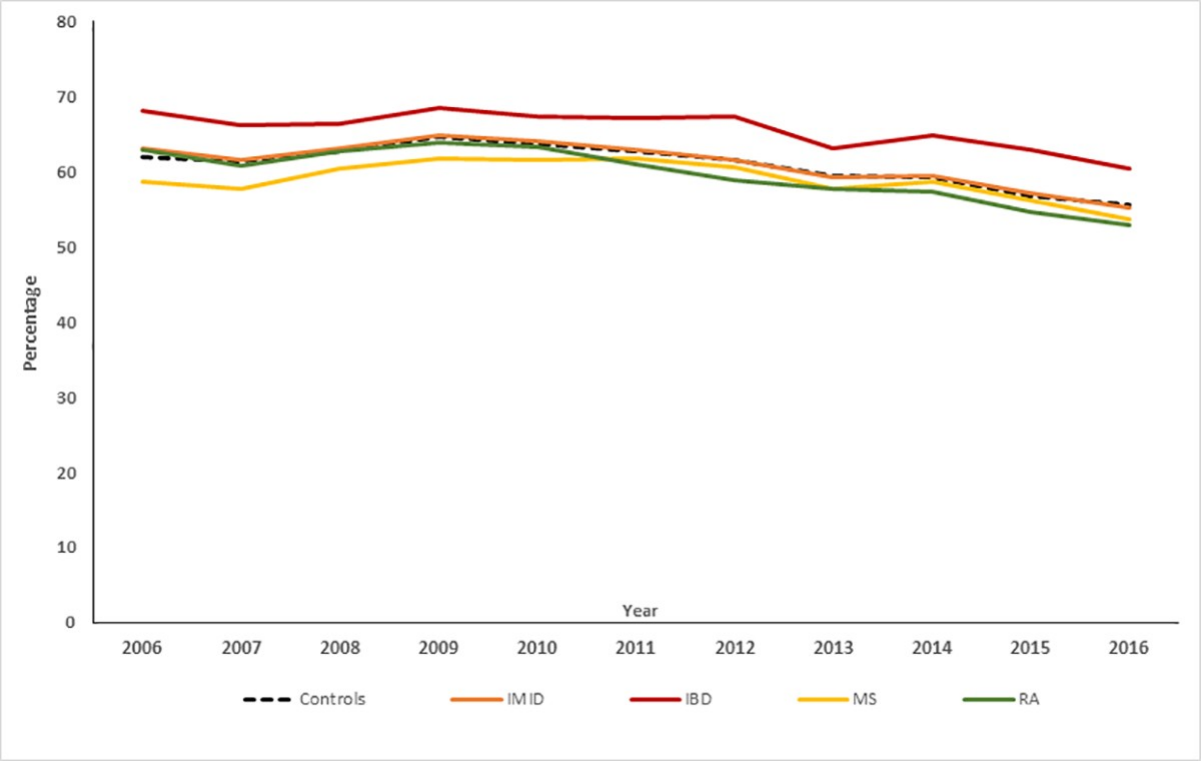
Mammography participation rates for 2006–2016 age-standardized to 2010 population.

In 2016, 55.8% (95%CI: 54.6, 56.9%) of the IMID cohort underwent a mammogram, which did not differ from the matched cohort (56.3%; 95%CI: 55.8, 56.9%) (risk difference [RD] 0.58%; 95%CI: -0.70%, 1.86%, Fig 1). After adjustment for active mood/anxiety disorder, mammography use did not differ between the IMID and matched cohorts (RD 0.4%; 95%CI: -0.5, 1.2%). After adjustment for IMID status, as compared to persons without an active mood/anxiety disorder, those with an active mood/anxiety disorder participated less in mammography (RD -2.5%; 95%CI: -4.0, -1.0%). Within persons, having an active rather than inactive mood/anxiety disorder was associated with slightly greater use of mammography (RD 0.6%; 95%CI: 0, 1.2%).

On multivariable analysis adjusting for age, socioeconomic status, region, physical comorbidities, immune therapy and IMID-specific procedures, the IMID cohort was less likely to undergo mammography than the matched cohort. However, the magnitude of the effect was quite small (Table 2). As compared to persons without a mood/anxiety disorder, a lower proportion of those with mood/anxiety disorder underwent mammography. Within individuals, variations in having an active mood/anxiety disorder rather than an inactive mood/anxiety disorder were not associated with any difference in the use of mammography. We did not observe any interactions between cohort and mood/anxiety disorder status on use of mammography.

**Table 2:** Adjusted^a^ association of immune-mediated inflammatory disease (IMID), any mood or anxiety disorder (MAD) and mammogram use (proportion; 95% confidence interval).

Variable	IMID	IBD	MS	RA
Cohort effect^b^	**-0.010 (-0.019, -0.001)**	**0.029 (0.010, 0.048)**		**-0.015 (-0.027, -0.002)**
When between-person effect of MAD absent			**-0.052 (-0074, -0.031)**	
When between-person effect of MAD present			**-0.048 (-0.084, -0.011)**	
When within-person effect of MAD absent			**-0.052 (-0.074, -0.031)**	
When within-person effect of MAD present			**-0.048 (-0.084, -0.011)**	
Between-person effect of MAD	**-0.021 (-0.036, -0.007)**	**-0.037 (-0.065, -0.008)**		**-0.023 (-0.043, -0.003)**
Within-person effect of MAD	0.006 (-0.001, 0.012)	0.006 (-0.006, 0.018)		0.007 (-0.002, 0.015)
Among MS cases				
Between-person effect of MAD			0.062 (-0.001, 0.125)	
Within-person effect of MAD			0.005 (-0.021, 0.031)	
Among Matches				
Between-person effect of MAD			-0.022 (-0.056, 0.012)	
Within-person effect of MAD			0.00 (-0.014, 0.014)	

IBD = inflammatory bowel disease, MS = multiple sclerosis, RA = rheumatoid arthritis, IMID = immune-mediated inflammatory disease, combining IBD, MS and RA cohorts; MAD = mood or anxiety disorder

a-Adjusted for age, region, socioeconomic status, year of diagnosis, disease duration, use of immune therapy, physical comorbidities, and IMID-specific procedures

b- Association of cohort status (IMID vs. not IMID) with mammography use; 95% confidence intervals that do not include zero are statistically significant at α = 0.05 as indicated by boldface values.

### Mammograms in individual IMID cohorts

The findings differed across IMID. The IBD cohort used mammography more than its matched cohort. In contrast, mammography use was lower in the MS and RA cohorts than in their matched cohorts; the magnitude of this effect was greater for MS than for RA (Table 2). In IBD and RA, a lower proportion of individuals with an active mood/anxiety disorder underwent mammography than those without an active mood/anxiety disorder. We did not observe within-person effects of an active mood/anxiety disorder, nor did we observe any interactions between cohort and mood/anxiety disorder status on mammography use.

In MS we observed a positive interaction between cohort and active mood/anxiety disorder status (8.4%; 95%CI: 1.2, 15.5%); among persons with MS, having an active mood/anxiety disorder was associated with more subsequent mammography use, whereas having an active mood/anxiety disorder was not associated with any differences in mammography use in persons without MS (Table 2).

### Pap tests in IMID cohort

**Fig 2:**
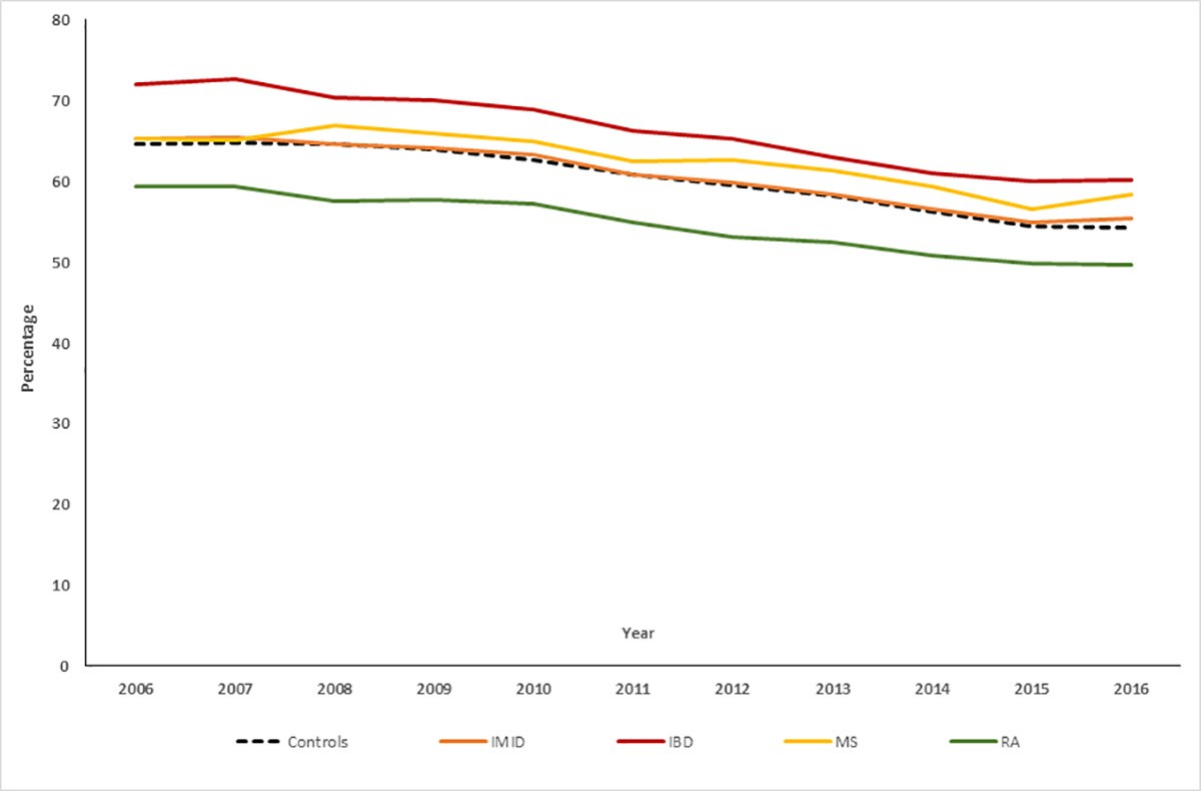
Pap testing frequencies for 2006–2016 age-standardized to 2010 population.

In 2016, 49.7% (95%CI: 48.8, 50.7%) of the IMID cohort and 50.1% (95%CI: 49.7, 50.6%) of the matched cohort had undergone a Pap test within the previous three years; this did not differ between cohorts (risk difference [RD] 0.40%; 95%CI: -0.64%, 1.44%, Fig 2). After adjustment for active mood/anxiety disorder, Pap testing was slightly less frequent in the IMID cohort than in the matched cohort (RD -0.9%; 95%CI: -1.6%, -0.3%). As compared to persons without an active mood/anxiety disorder, those with an active mood/anxiety disorder participated more in Pap testing (RD 8.3%; 95%CI: 7.1, 9.5%). Within persons, having an active rather than inactive mood/anxiety disorder was associated with a slightly greater frequency of Pap testing (RD 0.6%; 95%CI: 0.1, 1.1%).

On multivariable analysis adjusting for age, socioeconomic status, region, physical comorbidities, immune therapy and IMID-specific procedures, we observed a negative interaction between cohort and mood/anxiety disorder status (between persons) on Pap testing (RD -5.2%; 95%CI: -8.1, -2.3%). Therefore, members of the IMID cohort without an active mood/anxiety disorder did not differ from members of the matched cohort with respect to Pap testing (Table 3).

**Table 3:** Adjusted^a^ association of immune-mediated inflammatory disease (IMID), any mood or anxiety disorder (MAD) and pap testing (proportion; 95% confidence interval).

Variable	IMID	IBD	MS	RA
Cohort effect^b^			**-0.034 (-0.049, -0.019)**	
When between-person effect of MAD absent	-0.002 (-0.010, 0.007)	**0.043 (0.028, 0.059)**		**-0.019 (-0.030, -0.007)**
When between-person effect of MAD present	-0.054 (-0.008, -0.028)	**-0.060 (-0.107, -0.013)**		**-0.026 (-0.047, -0.005)**
When within-person effect of MAD absent	-0.002 (-0.01, 0.007)	**0.043 (0.028, 0.059)**		**-0.019 (-0.030, -0.007)**
When within-person effect of MAD present	-0.011 (-0.025, 0.003)	**0.033 (0.007, 0.059)**		**-0.078 (-0.117, -0.038)**
Between-person effect of MAD			**0.070 (0.046, 0.094)**	
Within-person effect of MAD			**0.014 (0.005, 0.023)**	
Among IMID cases				
Between-person effect of MAD	**0.037 (0.011, 0.062)**	-0.024 (-0.068, 0.021)		**0.038 (0, 0.076)**
Within-person effect of MAD	0.001 (-0.009, 0.011)	0.002 (-0.017, 0.020)		-0.003 (-0.018, 0.013)
Among Matches				
Between-person effect of MAD	**0.089 (0.075, 0.10)**	**0.079 (0.054, 0.105)**		**0.097 (0.077, 0.117)**
Within-person effect of MAD	**0.01 (0.005, 0.016)**	**0.012 (0.002, 0.022)**		0.005 (-0.003, 0.013)

IBD = inflammatory bowel disease, MS = multiple sclerosis, RA = rheumatoid arthritis, IMID = immune-mediated inflammatory disease, combining IBD, MS and RA cohorts; MAD = mood or anxiety disorder

a-Adjusted for age, region, socioeconomic status, year of diagnosis, disease duration, use of immune therapy, physical comorbidities, and IMID-specific procedures

b- Association of cohort status (IMID vs. not IMID) with Pap testing; 95% confidence intervals that do not include zero are statistically significant at α = 0.05 as indicated by boldface values

However, members of the IMID cohort with an active mood/anxiety disorder were significantly less likely to undergo Pap testing than members of the IMID cohort without an active mood/anxiety disorder. Within the IMID cohort, individuals with an active mood/anxiety disorder were less likely to undergo Pap testing than individuals without an active mood/anxiety disorder. The magnitude of this effect was more than 50% smaller than in the matched cohort. Within an individual, changes in mood/anxiety disorder status from inactive to active were associated with very small increases in Pap testing.

### Pap tests in individual IMID cohorts

Findings differed across the individual IMID cohorts. The frequency of Pap testing was higher in the IBD cohort than in its matched cohort in the absence of an active mood/anxiety disorder, but the frequency of Pap testing was lower in the IBD cohort than in the matched cohort in the presence of an active mood/anxiety disorder. As compared to an inactive mood/anxiety disorder, having an active mood/anxiety disorder was not associated with differences in the frequency of Pap testing within the IBD cohort, however, it was associated with more Pap testing in the matched cohort.

The frequency of Pap testing was lower in the MS cohort than in its matched cohort (Table 3). Having an active mood/anxiety disorder was associated with a greater frequency of Pap testing, as were within-person changes from inactive to active mood/anxiety disorder status.

Regardless of mood/anxiety disorder status, the frequency of Pap testing was lower in the RA cohort than in its matched cohort. We observed a negative interaction between RA status (yes vs. no) and mood/anxiety disorder status (between persons) on Pap testing (RD -5.9%; 95%CI: -10.2, -1.6%). This meant that although having an active mood/anxiety disorder was associated with increased Pap testing in the matched cohort, the magnitude of this effect was less in the RA cohort, such that the joint effect of RA and an active mood/anxiety disorder was no significant increase in Pap testing.

## Discussion

In this population-based study, we examined the joint effects of IMID and psychiatric comorbidity on cervical and breast cancer screening. Contrary to our hypothesis, the effects of IMID and comorbid mood/anxiety disorder on cancer screening were not uniform. We found that overall, participation in screening was relatively low and having an IMID was associated with slightly (1%) lower use of mammography; however, this reflected an average effect of greater use of mammography in the IBD cohort (2.9%) and lower use in the MS (4.8–5.2%) and RA (1.5%) cohorts. Within the IBD, MS and RA cohorts, having an active mood/anxiety disorder was associated with more mammography use than having an inactive mood/anxiety disorder. Overall, the MS and RA cohorts were less likely to undergo Pap testing than their matched cohorts; however, within the MS cohort having an active mood/anxiety disorder was associated with greater Pap testing. In the RA cohort, having an active mood/anxiety disorder was associated with greater Pap testing but this was almost fully counterbalanced by the lower testing associated with having RA. In the absence of an active mood/anxiety disorder, the IBD cohort was more likely to undergo Pap testing than its matched cohort, but the opposite was true in the presence of an active mood/anxiety disorder. Notably, findings in the IBD cohort generally diverged from those in the MS and RA cohorts. Given the reported barriers to screening among women with disabilities [30], this may reflect the greater level of physical impairment which occurs in MS and RA than in IBD, but we were unable to evaluate this possibility because this information is not captured in administrative data. It also may reflect that persons with IBD are at a two-fold increased risk of colorectal cancer and the need for surveillance colonoscopies is typically discussed early in management planning [31]. Hence, IBD patients are introduced to cancer prevention strategies early in their diagnosis and often at young ages.

In Canada, the target participation rate for screening mammograms among women aged 50–69 years is ≥70% within 30 months [32]. We found that participation rates were below this target in all cohorts studied, consistent with prior reports that mammography participation was 54% across Canada in 2012 [32]. Similarly, participation rates for cervical cancer screening were below the target of ≥80% within 42 months [33]. Prior studies which examined the frequency of mammograms and Pap tests in IMID populations were mostly cross-sectional, and evaluated smaller, selected populations. Findings have been variable. A study in Manitoba over the period 2002 to 2008 found that 47% of women with IBD aged 50–69 years had mammograms, similar to matched controls [34]. Fifty-four percent of women with IBD aged 18–69 years underwent Pap tests, which was less than women without IBD, but this study did not account for comorbidity. In contrast, a study at Kaiser-Permanente in the United States reported that 93% of women with IBD underwent Pap tests, exceeding the percentage of women without IBD who did [35]. In Denmark, women with Crohn’s Disease underwent Pap tests as often as women without IBD, but women with Ulcerative Colitis underwent Pap tests more often [36]. Studies in the United States and United Kingdom have reported that 48% to 77% of women with MS undergo mammograms over intervals ranging from 1 to 5 years, while 41.8% to 85% undergo Pap tests [8, 9, 37, 38]. The lowest participation rates have been observed among women with severe disability [9]. However, none of these studies have included comparisons to non-MS populations. In the RA population, participation rates for mammograms vary from 66% to 94%, and for Pap smears from 70–94%, with particularly high participation rates reported by participants in the Nurses Health Study cohorts [3, 39]. Nurses with RA were more likely to receive Pap tests and mammograms than nurses without RA; however this study did not account for psychiatric comorbidity [40]. Among women aged ≥65 years with Medicare insurance, women with RA had lower odds of undergoing a mammogram than women with osteoarthritis, but this study did not account for psychiatric comorbidity [41].

The association between having an active mood/anxiety disorder and screening differed for mammograms and Pap tests, and also differed across IMIDs. The literature regarding the impact of psychiatric illness on preventive care, including cancer screening is similarly variable, with some studies citing improved uptake of screening, others no effect, and others worsened uptake [42]. In the Canadian National Population Survey the presence of major depressive disorder [43], as identified using a short form version of the World Health Organization Composite International Diagnosis Interview (CIDI) [44], was not associated with differences in the frequency of mammograms or Pap tests. In contrast, in Ontario, Canada, a study which linked responses from the 2002 Canadian Community Health Survey to administrative data found that the presence of major depressive disorder or elevated depressive symptoms were associated with lower screening rates for mammograms and pap tests [17]. Among women with chronic disease, depression has been associated with lower participation rates for Pap tests and mammograms. Among 5,869 participants in the Behavioral Risk Factor Surveillance Survey with self-reported stroke, those with elevated symptoms of depression were less likely to report undergoing a mammogram or Pap test than those without symptoms of depression [45]. Similarly, among women with diabetes, those with elevated symptoms of depression were less likely to report receiving a mammogram in the last two years than those without symptoms of depression, but the frequency of Pap tests did not differ [46]. Given our findings that mood/anxiety disorders are associated with differences in cancer screening in three different IMID and for two different cancer screening tests, and given the existing literature regarding the effects of depression on screening in the general population and other chronic disease populations as described, psychiatric disorders do appear to be an important factor to consider when evaluating participation in cancer screening. Notably, the effects of psychiatric comorbidity are not uniform across chronic diseases.

Limitations of this population-based study should be considered. We used administrative data, thus we could not account for IMID disease characteristics, including physical disability, although we accounted for IMID-related surgery and use of immune therapies. We could not distinguish between mammograms used for diagnostic purposes rather than screening purposes, but diagnostic mammograms are much less common than screening mammograms. We conducted this study in only one province, but participation rates in mammograms and Pap smears are similar across provinces in Canada [32, 33]; however, our findings may not generalize to other health systems. Although we identified comorbid mood/anxiety disorder using validated case definitions, this likely does not represent the full burden of mood/anxiety disorder as encounters with non-physician providers are not captured in our data. We considered important potential confounders such as age, socioeconomic status, physical comorbidity and geography but we were unable to account for health behaviors such as such smoking, and obesity. We were also unable to account for other factors that may influence participation in cancer screening such as risk tolerance, health literacy or education regarding cancer screening.

Having an IMID and an active mood/anxiety disorder influence participation in cancer screening among women. MS and RA are associated with less participation in breast and cervical cancer screening. Given these lower participation rates, structured approaches to improving screening in these populations should be tested. The effects of psychiatric comorbidity on participation in cancer screening vary across IMID and are complex; an adverse effect of mood/anxiety disorder on cancer screening should not be presumed in individuals with IMID. For clinicians this highlights the importance of an individualized approach to care. Future studies aimed at improving screening rates in IMID populations should account for psychiatric comorbidity.

## Supporting information

S1 Table Tariff and procedure codes to identify surgeries for inflammatory bowel disease. (DOCX)

S2 Table Diagnostic and procedure codes to identify joint-related procedures for rheumatoid arthritis. (DOCX)

S3 Table Immune therapies for immune-mediated inflammatory disease. (DOCX)
