# Supplementary material for: Effect of comorbid mood and anxiety disorders on breast and cervical cancer screening in immune-mediated inflammatory disease
Source: PLoS One. 2021 Aug 5;16(8):e0249809. doi: 10.1371/journal.pone.0249809 (PMC8341605; doi:10.1371/journal.pone.0249809)
Supplement: S1 Table — (DOCX) [file pone.0249809.s001.docx]

S1 Table. Tariff and procedure codes to identify surgeries for inflammatory bowel disease

| **Procedure** | **Physician Claim Tariff Codes** |
| --- | --- |
| Intestinal suture | 3221, 3223 |
| Colostomy | 3195 |
| Colectomy | 3179 |
| Colectomy, proctectomy | 3180, 3181, 3182, 3183, 3184 |
| Proctosigmoidectomy | 3286 |
| Proctosigmoidectomy, protectomy | 3288, 3289, 3290 |
| Rectal surgery | 3301 |
| Small bowel resection | 3201, 3174 |
| Ileostomy | 3193 |
|  | **Hospital Procedures** |
| Incision, excision & anastomosis of intestine | 45, excluding 451 & 452 diagnostic procedures  1NM – colon + 59, 87, 89, 92 excision |
