# Supplementary material for: Effect of comorbid mood and anxiety disorders on breast and cervical cancer screening in immune-mediated inflammatory disease
Source: PLoS One. 2021 Aug 5;16(8):e0249809. doi: 10.1371/journal.pone.0249809 (PMC8341605; doi:10.1371/journal.pone.0249809)
Supplement: S2 Table — (DOCX) [file pone.0249809.s002.docx]

S2 Table. Diagnostic and procedure codes to identify joint-related procedures for rheumatoid arthritis

| **Procedure** | **ICD-9-CM code** | **CCI code** |
| --- | --- | --- |
| Hip replacement (incl. Partial, total, revisions) | 81.50, 81.51, 81.53, 81.52 | 1.VA.53.LA-PN^^ or 1.VA.53.PN-PN^^  1.VA.53.LA-PM^^ or 1.VA.53.PN-PM^^ |
| Knee replacement | 81.54, 81.55 | 1.VG.53.LA-PN or 1.VG.53.LA-PP |
| Elbow replacement | 81.84 | 1.TM.80.^^ |
| Shoulder replacement | 81.80, 81.81, 81.83 | 1.TA.53.^^ |
| Wrist fusion | 81.25, 81.26 | 1.UB.75.^^, 1.UG.75.^^ |
| Ankle fusion | 81.1 | 1.WA.75.^^ |
| Foot joint fusion | 81.1 | 1.WE.75.^^ |
| Foot joint replacement | 81.57 | 1.WJ.53.^^ |
| Cervical spine fusion | 81.01, 81.02, 81.03 | 1.SA.75.^^ |
| Hand joint replacement | 81.7 | 1.UC.53.^^, 1.UC.55. ^^ |

ICD-9-CM = International Classification of Diseases, 9^th^ revision, Clinical Modification; CCI = Canadian Classification of Intervention code. ^ CCI codes are 10 characters long and broken into components. The first number in the code followed by 2 letters indicates anatomical location or development stage. Subsequent codes provide more specific information about the intervention. The ^ indicates that ll of the codes with more specific indicators were included.
