# Supplementary material for: Effect of comorbid mood and anxiety disorders on breast and cervical cancer screening in immune-mediated inflammatory disease
Source: PLoS One. 2021 Aug 5;16(8):e0249809. doi: 10.1371/journal.pone.0249809 (PMC8341605; doi:10.1371/journal.pone.0249809)
Supplement: S3 Table — (DOCX) [file pone.0249809.s003.docx]

S3 Table. Immune therapies for immune-mediated inflammatory disease

| **Treatment**  **Disease** | **Inflammatory Bowel Disease** | **Multiple Sclerosis** | **Rheumatoid Arthritis** |
| --- | --- | --- | --- |
| **Corticosteroids^1^** | Methylprednisolone (H02AB04)  Prednisolone (H02AB06)  Prednisone (H02AB07)  Budesonide  Hydrocortisone (enema) | Methylprednisolone (H02AB04)  Prednisolone (H02AB06)  Prednisone (H02AB07) | Methylprednisolone (H02AB04)  Prednisolone (H02AB06)  Prednisone (H02AB07)  Triamcinolone (H02AB08)  Cortisone (H02AB10)) |
|  |  |  |  |
| **Anti-inflammatory or**  **Immunomodulatory therapies^2^** | 5-ASA (A07EC02, A07EC03)  Sulfasalazine (A07EC01) | Glatiramer acetate (L03AX13)  Interferon-beta 1a (L03AB07)  Interferon-beta 1b (L03AB08)  Dimethyl fumarate (N07XX09)  Teriflunomide (L04AA31)  Peg interferon-beta (L03AB13) | Sulfasalazine (A07EC01)  Sodium aurothiomalate (M01CB01)  Auranofin (M01CB03)  Aurothioglucose (M01CB04)  Penicillamine (M01CC01)  Hydroxychloroquine (P01BA02) |
| **Traditional immunosuppressive therapies^3^** | Azathioprine (L04AX01)  Methotrexate (L04AX03)  6-mercaptopurine (L01BB02)  Cyclosporine (L04AA01)  Tacrolimus (L04AD02) | Azathioprine (L04AX01)  Methotrexate (L04AX03)  Mitoxantrone (L01DB07)  Cyclophosphamide (L01AA01) | Azathioprine (L04AX01)  Methotrexate (L04AX03)  Cyclophosphamide (L01AA01)  Cyclosporine (L04AA01)  Leflunomide (L04AA13) |
|  |  |  |  |
| **Novel therapies/ Biologics^3^** | Infliximab (L04AA12)  adalimumab (L04AA17)  Golimumab (L04AB06)  Ustekinumab (L04AC05)  Vedolizumab (L04AA33) | Natalizumab (L04AA23)  Fingolimod (L04AA27)  Alemtuzumab (L04AA34)  Cladribine^4^ (L04AA40)  Ocrelizumab^4^ (L04AA36) | Infliximab (L04AA12)  adalimumab (L04AA17)  Etanercept (L04AA11)  Anakinra (L04AA14)  Rituximab (L01XC02)  Abatacept (L04AA24)  Tocilizumab (L04AC07)  Tofacitinib (L04AA29)  Golimumab (L04AB06)  Certolizumab (L04AB05) |

1. Corticosteroids were not included as immune therapies for MS because they are not used as an chronic therapy, but exclusively for episodic treatment of relapses. 2- Therapies in this row in the MS column were considered first-line. 3- Therapies in this row in the MS column were considered second-line. 4- Not available during the study period.
